# Chasing the conversation: Autistic experiences of speech perception

**DOI:** 10.1177/23969415221077532

**Published:** 2022-02-24

**Authors:** Alexandra Sturrock, Hannah Guest, Graham Hanks, George Bendo, Christopher J Plack, Emma Gowen

**Affiliations:** Division of Human Communication, Development and Hearing, School of Health Sciences, 5292 The University of Manchester, Manchester, UK; Manchester Centre for Audiology and Deafness, School of Health Sciences, The University of Manchester, Manchester, UK; Department of Psychology, 7315 The University of Sheffield, Sheffield, UK; Department of Physics and Astronomy, UK ALMA Regional Centre Node, Jodrell Bank Centre for Astrophysics, 5292 The University of Manchester, Manchester, UK; Manchester Centre for Audiology and Deafness, School of Health Sciences, The University of Manchester, Manchester, UK; Division of Neuroscience and Experimental Psychology, School of Biological Sciences, 5292 The University of Manchester, Manchester, UK

**Keywords:** Autism spectrum, autistic, speech perception, auditory processing disorder, hyperacusis

## Abstract

**Background and aims:**

Humans communicate primarily through spoken language and speech perception is a core function of the human auditory system. Among the autistic community, atypical sensory reactivity and social communication difficulties are pervasive, yet the research literature lacks in-depth self-report data on speech perception in this population. The present study aimed to elicit detailed first-person accounts of autistic individuals’ abilities and difficulties perceiving the spoken word.

**Methods:**

Semi-structured interviews were conducted with nine autistic adults. The interview schedule addressed interviewees’ experiences of speech perception, factors influencing those experiences, and responses to those experiences. Resulting interview transcripts underwent thematic analysis. The six-person study team included two autistic researchers, to reduce risk of neurotypical ‘overshadowing’ of autistic voices.

**Results:**

Most interviewees reported pronounced difficulties perceiving speech in the presence of competing sounds. They emphasised that such listening difficulties are distinct from social difficulties, though the two can add and interact. Difficulties were of several varieties, ranging from powerful auditory distraction to drowning out of voices by continuous sounds. Contributing factors encompassed not only features of the soundscape but also non-acoustic factors such as multisensory processing and social cognition. Participants also identified compounding factors, such as lack of understanding of listening difficulties. Impacts were diverse and sometimes disabling, affecting socialising, emotions, fatigue, career, and self-image. A wide array of coping mechanisms was described.

**Conclusions:**

The first in-depth qualitative investigation of autistic speech-perception experiences has revealed diverse and widespread listening difficulties. These can combine with other internal, interpersonal, and societal factors to induce profound impacts. Lack of understanding of such listening difficulties – by the self, by communication partners, by institutions, and especially by clinicians – appears to be a crucial exacerbating factor. Many autistic adults have developed coping strategies to lessen speech-perception difficulties or mitigate their effects, and these are generally self-taught due to lack of clinical support.

**Implications:**

There is a need for carefully designed, adequately powered confirmatory research to verify, quantify, and disentangle the various forms of listening difficulty, preferably using large samples to explore heterogeneity. More immediate benefit might be obtained through development of self-help and clinical guidance materials, and by raising awareness of autistic listening experiences and needs, among the autistic community, communication partners, institutions, and clinicians.

## Introduction

**Figure 1.**
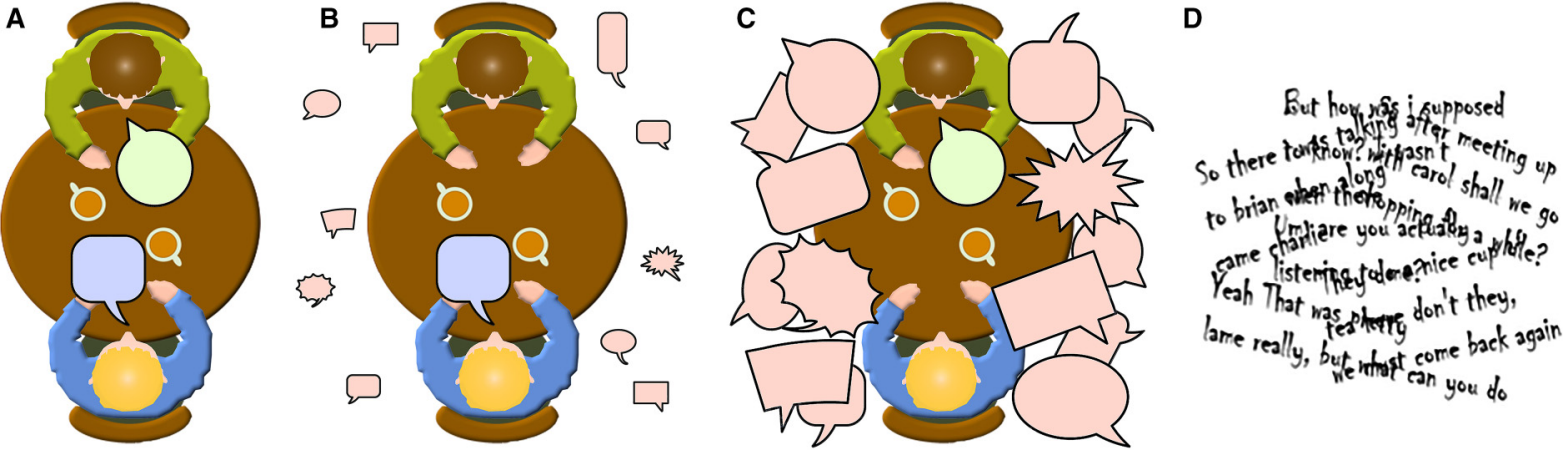
An illustration by GH of the challenges posed to some autistic listeners by common social listening environments, which he dubbed ‘The café effect’. (A) The person in blue is autistic, the person in green neurotypical. The pair attempt conversation in the presence of background noise which is not loud but is composed of multiple voices. (B) When blue speaks, green has some awareness of the background voices, but is not troubled by them. (C) When green speaks, blue experiences significant interference from the background voices. (D) The resulting jumble of sounds makes comprehension challenging.

The autistic population is a sizeable minority; recent evidence suggests that 2.2% of adults are autistic (Dietz et al., 2020). Improved understanding of the traits and needs of this community is essential, so that its members can participate fully in society. In July 2019, an Expert by Experience Advisory Group convened by Autism@Manchester set out to identify autism research priorities. Two of its members – authors GB and GH – independently proposed speech perception in real-world listening environments. Figure 1 presents an illustration created by GH to convey the challenges posed by some common social listening environments, a phenomenon he dubbed ‘The Café Effect’.

Contemporary diagnostic criteria recognise atypical sensory reactivity as a core autistic trait (American Psychiatric Association, 2013), and personal reports have long indicated that speech perception may diverge from that of the neurotypical population (Grandin, 1992). However, the existence and nature of speech-perception anomalies remain unclear; researchers seeking experimental evidence have used manifold approaches and measures, often in very small samples, yielding inconsistent results. Speech-perception tasks used in children have tended to rely upon single-word or single-syllable stimuli, while those in adults have used sentences. The background sounds used in which the speech is embedded have ranged from simple white noise to speech ‘babble’. With a couple of exceptions (Foxe et al., 2015; Stevenson et al., 2018), most studies have been underpowered, and most reported effects would not survive correction for multiple comparisons. At our most optimistic, we might take the assembled results to indicate inconsistent evidence for word-perception-in-noise deficits in autistic children (Foxe et al., 2015; Groen et al., 2009; Irwin et al., 2011; Stevenson et al., 2018) and uncertain trends towards sentence-perception-in-noise deficits in autistic adults and adolescents (Alcántara et al., 2004; Dunlop et al., 2016; Schelinski & von Kriegstein, 2020; Schelinski et al., 2014). For example, if we consider the auditory data in the two largest of these studies, autistic participants exhibited small deficits for word recognition in noise that would not survive correction for multiple comparisons in most age-groups (Foxe et al., 2015) and no deficits for word recognition in babble (Stevenson et al., 2018).

What is largely absent from the research literature is a more fundamental approach: asking autistic people to describe in detail their speech-perception experiences. First-hand qualitative data on sensory experiences do exist (Ashburner et al., 2013; Jones et al., 2003; Kirby et al., 2015; Robertson & Simmons, 2015, 2018), but none focus on audition specifically, meaning that deep data are lacking. We argue that such self-report data are essential, for several reasons. First, researching autistic speech perception without ever asking autistic people what they perceive is an odd thing to do. It is also arguably disempowering; many members of the autistic community are capable of reporting and reflecting upon their auditory experiences with great clarity, offering an experiential perspective devoid of neurotypical misinterpretation. Access to such data would likely increase the efficiency and validity of quantitative research, guiding auditory scientists’ selection of hypotheses and methods and targeting community priorities. Current lack of data on the impact of speech-perception difficulties also denies society an understanding of the importance of these issues and of what resources should be devoted to them. Finally, autistic insight on difficult speech-perception experiences might yield immediate benefit, by informing both the autistic community and neurotypical allies on strategies and measures (by the individual, by communication partners, and by institutions) that might reduce negative impact on autistic individuals.

The aims of the present study were to:

- Explore the nature of speech-perception abilities and difficulties in a small sample of autistic adults
- Explore the impacts of and responses to speech-perception anomalies in a small sample of autistic adults
- Achieve the above via genuine collaboration with autistic researchers

A semi-structured interview approach was selected, with resulting transcripts interrogated via thematic analysis. Autistic members of the project team were integral in developing the interview materials, analysing the data, and preparing the present report, including all figures.

## Material and methods

### Participants

Nine autistic adults took part, aged 19 to 38 years (median = 28), four of whom identified as male and five as female. Participants were British (n = 5) or North American (n = 4) and native speakers of English. All were recruited via digital postings on social networks and internet message boards. Participants were given the option to self-identify as ‘diagnosed as autistic’ or ‘seeking a diagnosis’; all nine were ‘diagnosed as autistic’ and no proof of diagnosis or further information on diagnostic methods were sought. None had ever been diagnosed with hearing loss. Data on race and socio-economic status were not sought. No formal constraint was placed on cognitive abilities, nor were they measured, but due to the study's procedures for recruitment and data collection, it is likely that self-selection biased the sample to individuals without intellectual disability.

### Interview schedule

A qualitative interview schedule (see SM1) was developed by an autism researcher (AS), an auditory researcher (HG), and an autistic researcher (GH). The schedule aimed to explore participants’ views and experiences on (a) speech-perception abilities/difficulties and (b) responses to any speech-perception difficulties. (Feedback on hearing research was also sought by the interviewer at the close of the interview but was not included in the thematic analysis.) Each area was addressed via three-four open-ended questions. Each area also contained the option for six-eight follow-up prompts, each aiming to elicit a response on a specific sub-topic if such a response was not provided unprompted by the participant. To reduce the influence of researcher bias, care was taken to phrase initial questions neutrally (‘How easy or difficult do you find …’) and follow-up questions on speech-perception difficulties were not posed unless the interviewee had mentioned experiencing such difficulties.

Note that the phrase ‘speech perception’ was considered excessively technical and was not used during interviews. Instead, the interviewer explained at the outset that she wished to learn about the interviewee's ‘experiences of **hearing speech** (i.e., hearing what people are saying)’ and subsequently reinforced the notion that *speech* hearing was the focus of the interview (see SM1). Throughout the interview, the colloquial terms ‘hearing’ and ‘listening’ were used, the latter denoting only those experiences involving *deliberate attention* to speech, the former defined more broadly, as passive or active perception of speech stimuli. For the sake of simplicity and brevity, the remainder of this paper will also use these terms, as defined here.

### Interview procedure

Each participant attended a single interview session lasting <1 h. All but one of the interviews were conducted via video link, with the remaining interview conducted in person on the University of Manchester. Five participants were interviewed by HG and four by AS. To minimise stress caused to participants, those interviewed via video link were given the choice as to whether to be viewed by the interviewer or to communicate solely via the audio channel. In practice, all chose to appear via video; the interviewer always appeared via video. Recordings of the interviews used for analysis were audio only. All procedures were approved by the Proportionate Review Ethics Panel at the University of Manchester.

### Data analysis

#### Analytical approach

The core analysis team comprised autism researcher AS, auditory researcher HG, and autistic researcher GH, each having equal responsibility for analysis. Interviews were transcribed verbatim, then coded into emergent themes via thematic analysis (Braun & Clarke, 2006). The team met via video link to discuss transcripts and themes on four occasions. Prior to each meeting, the members independently analysed one or more transcripts, producing notes that were subsequently shared with the team and formed the basis for group discussion. At each meeting, the notes of GH were given priority, to reduce the risk of neurotypical overshadowing of autistic voices (Milton, 2012). Consensus on emerging themes was sought on a point-by-point basis (Barker & Pistrang, 2005). During each meeting, the team drafted a document detailing their analysis decisions, which was distributed immediately after the meeting so that team members might review the emerging codes and themes for errors or misrepresentations. For transparency, evidence of any resulting alterations was retained in email trails and attached documents, as were all researchers’ independently created notes.

#### Theme generation, review, definition, and refinement

In the first round of analysis, all three team members considered the same single transcript (independent familiarisation and coding, followed by discussion and consensus-finding). This transcript yielded a rich array of 27 tentative subthemes, loosely grouped into three areas: speech-hearing anomalies and contributing factors, impacts of these anomalies, and coping mechanisms. Definition of main themes was deliberately and explicitly deferred to Meeting 2.

The second round of analysis incorporated three further transcripts, each considered by all three researchers, and yielded six main themes. In the third round, three further transcripts were considered, one by each researcher. Care was taken at the analysis meeting to allow sufficient discussion of all three. The fourth round of analysis comprised consideration of both remaining transcripts by all three researchers. At this late stage, data appeared largely saturated and modifications to the theme list were overwhelmingly organisational.

Final checking and minor reorganisation of the theme list was conducted via email. As part of this process, the team validated a subset of themes (the sub-themes within Main Theme 1) by reviewing all transcripts (one researcher per transcript) to determine code frequencies. This frequency check was found to validate the existing theme structure; each subtheme was raised by 3–7 (median = 6) interviewees. As an additional validation measure, the resulting final theme list and associated quotes were reviewed by autistic researcher GB and auditory scientist CP. These researchers judged the themes to be coherent, rich, and representative, but identified issues that our team has subsequently addressed: ambiguous language, quotes that lacked specificity when presented without context, and statements that needed to be softened or qualified.

## Results

**Figure 2.**
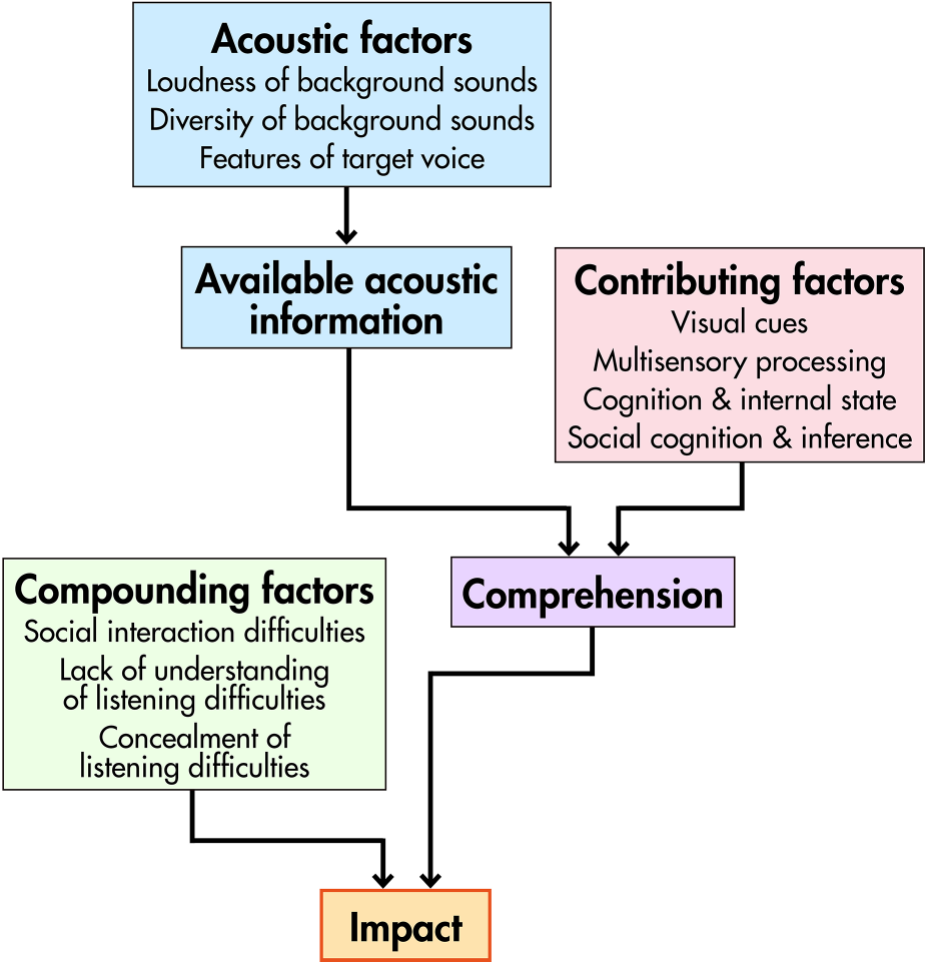
Relations between themes.

Interviews elicited rich and detailed accounts, with wide-ranging clinical and research implications. Six themes emerged, encompassing 28 subthemes, each with one or more tertiary themes. (Table 1 lists main themes and subthemes; Figure 2 schematises relations between themes; SM2 provides an expanded table including all 78 tertiary themes.) Because of the density of the data, detailed consideration of all tertiary themes in the main text is not feasible. Hence, we provide relatively simple descriptions of speech-perception anomalies, contributing factors, and compounding factors (Themes 1–4), followed by richer characterisations of impact and coping mechanisms (Themes 5 and 6).

**Table 1.** Main themes and subthemes.

**Main themes and subthemes**	
**1. Speech-perception anomalies** 1.1 Focusing on a voice amid background sounds 1.2 Distinguishing a voice from background sounds 1.3 Drowning out of a voice by background sounds 1.4 Orienting to a voice amid background sounds 1.5 Loudness discomfort and auditory overload 1.6 Acute hearing sensitivity	**2. Contributing factors (acoustic)** 2.1 Loudness of background sounds 2.2 Diversity of background sounds 2.3 Features of target voice
**3. Contributing factors (non-acoustic)** 3.1 Visual cues 3.2 Multi-sensory processing 3.3 Cognition and internal state 3.4 Social cognition and inference to support meaning	**4. Compounding factors** 4.1 Social interaction difficulties 4.2 Lack of understanding of listening difficulties 4.3 Concealment of listening difficulties
**5. Impact** 5.1 Social participation 5.2 Listening effort and listening-related fatigue 5.3 Emotion 5.4 Self-perception 5.5 (Perceived) impression made on others 5.6 Practical costs	**6. Coping mechanisms** 6.1 Self-awareness and self-advocacy 6.2 Developing auditory skills 6.3 Communication tactics 6.4 Managing the listening environment 6.5 Technology 6.6 Withdrawal/avoidance

### Theme 1: speech-perception anomalies

This theme draws together aspects of speech perception that participants identify as particularly challenging, particularly easy, or otherwise distinguishing their experiences from those of the neurotypical population. A wide array of phenomena are reported, with significant heterogeneity evident both between and within participants, yielding sufficient evidence to support six subthemes. Some subthemes appear entirely distinct from one another (e.g. 1.1 ‘*Focusing …*’ and 1.3 ‘*Drowning out …*’); others possess greater scope for overlap, and the descriptive challenges inherent in differentiating subtle auditory experiences mean that participants’ descriptions have not all been unambiguously categorised.

The first four subthemes concern difficulties in perceiving ‘target’ speech (speech to which an individual wishes to listen) when other sound sources are present. A strongly supported subtheme is 1.1: *Focusing on a voice amid background sounds.* Participants describe situations in which target and background sounds are perceptually distinct, but in which it is difficult or impossible to maintain auditory attention on the target voice. Distractor sounds are most often other voices, but can also be non-speech sounds (especially high-pitched or unpredictable sounds), and needn't be loud or numerous to grab the listener's involuntary attention:

*“it doesn't matter how loud that noise is, it will take me out of the conversation completely”*

By contrast, 1.2 *(Distinguishing a voice from background sounds)* involves difficulties ‘picking out’ a voice from a jumble of background voices or, less commonly, from non-speech backgrounds. Subtheme 1.3 (*Drowning out of a voice by background sounds)* refers to listening situations where distinguishing and focusing on a target voice are untenable, because the target speech is obscured by continuous background noise – usually loud and often low-pitched:

*“I don't really go to the movie theatre, because a lot of the time I don't understand what's going on (…) A lot of times the bass overtakes the rest of it, and you just hear this {makes rumbly noise}”*

Occasionally, this drowning-out effect can be used beneficially, to mask aversive sounds:

*“During the day [the loud fan is] handy because my neighbours have kids and they’re loud, but it’ll drown them out.”*

Subtheme 1.4 *(Orienting to a voice amid background sounds)* refers to problems determining who is talking (especially in environments with many talkers) and directing attention to that voice. Some individuals note an association between this phenomenon and disruption of visual cues (see also 3.1). In addition to potentially disordered speech perception, many participants report issues of *Loudness discomfort and auditory overload* (Subtheme 1.5). Of course, it is possible that some autistic individuals are discomfited by other aspects of noisy environments (e.g., crowds), which might plausibly be mistaken for loudness discomfort. However, some interviewees are explicit that this is an auditory phenomenon:

*“I’m not afraid of the crowd, I’m afraid of the **noises** of the crowd.”*

These experiences are reported as distressing in their own right but also as impacting listening ability indirectly via effects on emotional state. Distinct from this hypersensitivity to *loud* sounds, some participants report the ability to perceive very *faint* sounds, imperceptible to those around them (Subtheme 1.6: *Acute hearing sensitivity*). This can be beneficial, but can also lead to distraction or irritation.

### Theme 2: contributing factors (acoustic)

Participants identify a wide array of factors contributing to difficulty or ease of listening; those that impact the listener via *effects on sound* are drawn together in Theme 2. Unsurprisingly, many participants note that *Loudness* (Subtheme 2.1) is detrimental to their speech perception. Louder environments tend to cause problems with distinguishing, drowning out, and loudness discomfort, but problems with *focusing* on a target voice are less loudness-dependent. *Diversity of background sounds* (Subtheme 2.2) is also thought to play a strong role; the vast majority of participants indicate that the greater the variety of sound sources, the greater the challenge in distinguishing a target voice. Even in some ostensibly *single-source* listening environments, room size and reverberation can create sufficient acoustic complexity to be challenging. Number of concurrent talkers appears more multifaceted in its effects. Some participants find that the more conversations occurring in the listening environment, the greater the difficulty. Others note that even a single competing voice is problematic. One participant finds a single competing conversation most troubling of all, due to powerful auditory distraction:

*“if there's only two other people in the restaurant, I’m going to have a hard time not listening to what they’re saying. I’m like, ‘What you got going on? I’m not eavesdropping. What did you say?!’ (…) When there's enough people around it all just blends, but if it's just a couple of people, it gets awkward.”*

However, one point of universal agreement is strong preference for single-talker, one-to-one communication:

*“Even a group of just two people is so much more difficult than just one. It's strange, because I really … I like having all of my friends there, but I don't like having … even three-person conversations, I find it much more difficult.”*

Also important are *Features of the target voice* (Subtheme 2.3): clarity, speed, accent, and vocal pitch.

### Theme 3: contributing factors (non-acoustic)

The third theme draws together elements of the listening experience that impact speech perception, but not via effects on sound. *Visual cues* (Subtheme 3.1) are considered valuable, though not always sufficient to ensure comprehension in crowded environments. These cues are thought especially important for orienting to a new talker, though also for ongoing comprehension:

*“if someone's trying to start talking to me from behind, if I can't see them start the conversation, I will not pick up that they’re talking to me at all.”*

Given some participants’ difficulty with eye contact, other visual cues – such as lip movements – may be of greater benefit:

*“I do have trouble with eye contact and things, but that's not from my hearing, it's just that it makes me uncomfortable (…) but looking at their mouth I kind of focus and concentrate on that.”*

The majority of participants report effects of *Multi-sensory processing* (Subtheme 3.2) on speech perception (beyond the helpful visual cues noted above). Distraction by other sensory inputs (smell, heat, vision, pain) is common:

*“I was trying to listen to what the speaker was saying and someone near me, I don't know, who had this really strong like perfume or something (…) it was so distracting.”*

However, stimulation of other senses in preferred ways can sometimes enhance listening ability:

*“When I’m listening to something, I want to be like messing around with something in my hand, I want to have something to look at.”*

The factors affecting listening ability need not be external; effects of *Cognition and internal state* (Subtheme 3.3) are also commonly described. Some participants report distraction by thoughts and emotions. Motivation, fatigue, and attention levels seem also to influence speech comprehension. Finally, when coping in adverse listening environments, some participants report using *Social cognition and inference to support meaning* (Subtheme 3.4). This is more evident in female self-report, though the small sample size prevents firm conclusions as to whether this represents a genuine area of male-female difference:

“*I’m looking at other people's reactions, and try to pick out whatever words that I do hear and try to kind of infer what's being said or what's going on (…) so sometimes I’m a little late with my reaction ‘cos I’m not getting it right away.”*

### Theme 4: compounding factors

Whereas the factors above affect an individual's ability to understand what is being said, the factors grouped under Theme 4 do not. Instead, they modulate the *impact* of listening difficulties on the person's experience and functioning. Co-existing *Social interaction difficulties* (Subtheme 4.1) are described as greatly exacerbating the effects of difficult listening on the individual (and vice versa). Participants emphasise that listening difficulties are distinct from social difficulties in autistic people, but describe the two phenomena as having cumulative and interacting effects. *Lack of understanding of listening difficulties* (Subtheme 4.2) appears to be another crucial compounding factor:

*“It's a thing where if you’re in a big, crowded environment and you’re asking people, “Can we keep it down a little bit?” And everyone else is going, “Well it's just as hard for us” (…) but I don't think it affects them in the same way that it affects me! I think to them it's probably just an annoyance, whereas to me, it's affecting my entire evening.”*

Some participants note that this lack of understanding can be especially challenging when dealing with authority figures in education or employment. Lack of *self*-understanding of listening difficulties is an additional problem that several participants identify as having affected them in earlier life. They describe having felt perplexed and disheartened, especially after seeking clarity and support from clinical services, only to be discharged when standard audiological tests produce ‘normal’ results, without further investigations or advice. It is clear that lack of *clinical* understanding of listening difficulties is pivotal:

*“you’re told again and again, “No, no there's nothing wrong”, and you’re trying to work out, ‘Then why can't I hear someone? Why can't I have a normal conversation?’”*

Perhaps related to this lack of understanding is Subtheme 4.3: *Concealment of listening difficulties.* Whilst occasional guessing and pretending in a difficult listening environment may be adaptive, some participants report excessive concealment, sometimes backfiring via missed information, feelings of isolation, and anxiety around getting ‘caught out’.

### Theme 5: impact

This substantial theme encompasses the many and varied impacts of listening difficulties on our autistic participants. Prominent among them are impacts on *Social participation* (Subtheme 5.1). Listening difficulties are universally described as a barrier to full participation in common social environments, causing many interviewees to limit the duration, frequency, and type of socialising they engage in:

*“There's very large social impacts. Like, I don't go to parties, and that's like a place where a lot of people socialise. And if I were to go to a party, I can't understand people, so I can't effectively socialise, whether I go or not.”*

This is seen as a barrier to relationship building:

*“I would love to have friends, and be able to just go out and hang out go to a restaurants and sit and have drinks and chat and talk and that kind of social stuff, but I don't get that, and I really avoid that. I think I would like to have that in my life though.”*

Another universally reported impact is *Listening effort and listening-related fatigue* (Subtheme 5.1). Listening is reported as being highly effortful at times, draining the listener of the mental resources needed for comprehension, reflection, and retention of spoken information:

*“I do feel I have to put in a lot of effort to hear correctly. You don't have infinite attentional resources so if you’re spending time and additional effort trying to understand someone, and trying to scrape out whatever meaning you can from that two or three-second auditory memory, that's time you’re not spending trying to understand what they’re saying.”*

Effortful listening is seen as leading to growing fatigue and limited endurance, and also as diminishing the joy and increasing the cost of social participation:

*“It feels like work – holding a conversation in the pub feels like work. Cause it's too much going on. Not just the actual social element of it, which I often find difficult anyway. But the actual act of listening.”*

**Figure 3.**
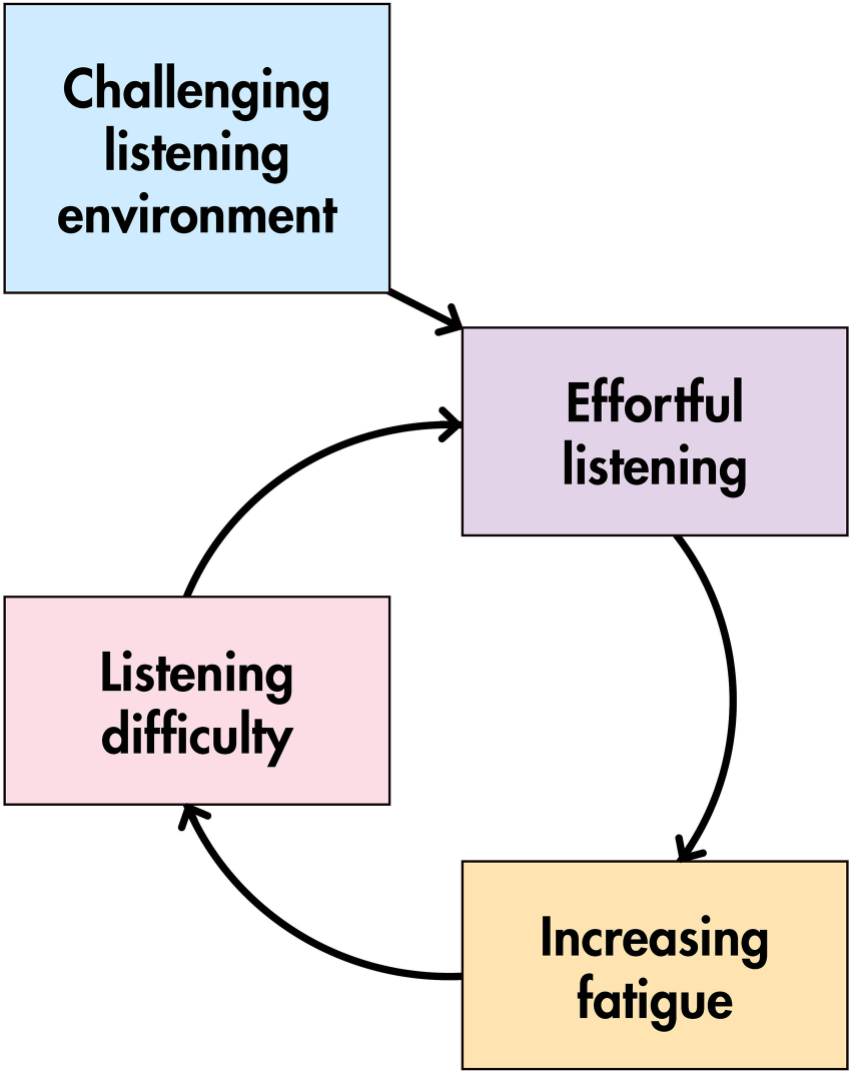
The effort-fatigue cycle.

Two participants also note an ‘effort-fatigue cycle’, whereby prolonged effortful listening induces fatigue, which in turn makes subsequent listening even more challenging (see Figure 3).

The majority of participants also explicitly mention the *Emotional impact* of listening difficulties (Subtheme 5.3). Many report negative emotions experienced in the moment, such as frustration, anxiety, and isolation:

*“It's very stressful because I’m missing out on stuff, and I know people are saying things, and my head starts going ‘you’re not reacting right’ or ‘you should have said something’ or ‘they asked you something and you don't know what they’re saying’”*

Emotional responses can be viscerally intense, associated with distress, pain, and nausea:

*“[Speaking of struggling to hear and perform well in a noisy call centre] I found I was terrified of going to work. I’d be almost vomiting in the street, walking to work like ‘OK, I just can't do this, I have to stop this.’”*

Even when not immediately adjacent to a difficult listening experience, people can experience persistent effects on emotions and well-being, such as dread, loneliness, and resentment. Given the above, it's perhaps unsurprising that most participants report negative effects on *Self perception* (Subtheme 5.4). Impacts on self-efficacy and self-esteem and feelings of resignation and self-blame are described:

*“I’ve like described feeling **broken** in relation to these things.”*

A closely related issue mentioned in most interviews is *Perceived impression made on others* (Subtheme 5.5). Almost all participants worry that listening difficulties cause their behaviour and character to be misunderstood by those around them. They suspect that difficulty hearing could be mistaken for stupidity, especially in work environments, or for apathy:

*“there's probably been times at parties with people think I’m ignoring them, or they’ve introduced themselves, and I’ve not even noticed them introducing themselves and come across as proper rude.”*

A final set of impacts concern *Practical costs* of listening difficulties (Subtheme 5.6). These include time (e.g., restriction to one-on-one meetings with friends rather than group socialising), money (e.g., noise-cancelling headphones), and costs to educational and occupational attainment:

*“It definitely hurt me in education. The first time I was in school I went to MIT. I got almost nothing out of lectures, I didn't understand what they were saying and I couldn't effectively take notes. It was all so distressing and I didn't understand why that was, or that that wasn't a universal experience.”*

### Theme 6: coping mechanisms

This final theme draws together behaviours – both deliberate and reflexive – that participants have used to cope with listening difficulties. *Self-awareness and self-advocacy* (Subtheme 6.1) emerges as a highly effective and empowering mindset, often developed in adulthood. The core of this perspective is understanding and accepting one's listening needs:

*“So a lot of it has just been training myself to pay attention, getting over just being shy about it, feeling embarrassed and feeling like there's something wrong with me, really helped a lot.”*

Expressing those needs takes various forms, and can be effective with or without disclosure of one's autistic identity:

*“I don't necessarily tell everyone that, ‘Oh, and by the way, I have received a diagnosis of autism.’ It is something which I treat, for the most part, as not particularly relevant. So, the way I’ll usually just explain to people is sort of a white lie, I’ll just say, ‘My hearing is a little bit off due to my years as a musician. So as a result, I can find it difficult to pick out voices unless I am pretty much right next to someone.’”*

*Developing auditory skills* (Subtheme 6.2) is also reported as valuable by some participants. For some, this process can be supported by positive life challenges:

*“It was really hard when I waited tables. I think that helped me a lot because I **have** to know what they’ve just said, you know – I **have** to know their order.”*

This ‘training’ process seems generally to be self-directed, and hence potentially unsystematic and uncertain; several participants suggest that more standardised guidance should be provided by professionals. Most interviewees also highlight the value of *Communication Tactics* (Subtheme 6.3) to aid listening. Central to this approach is the autistic listener asking communication partners to meet her communication needs, e.g., by positioning themselves appropriately, using clear speech, getting her attention before speaking, and clarifying content:

*“Now I know if I say ‘say again’ or ‘sorry?’ and they repeat themselves and I still can't understand them, I would be able to recognise to tell them ‘speak louder’ or ‘slow down’”*

For some participants, use of visual communication to supplement or replace oral communication is a constructive approach.

*“I also tend to look at people's mouths and kind of get some information there about what they’re saying. I do that a lot when I’m having a hard time”*

Where possible, seeking out skilled communication partners can be helpful. Most participants also benefit from deliberately *Managing the listening environment* (Subtheme 6.4). At its simplest, this can consist of choosing a preferable listening environment (typically quiet and calm, sometimes small and/or familiar):

*“In classes where we have group discussions, I’ll typically extract my group from the classroom and won't return.”*

There are reported benefits to restricting conversational group size (reflecting Subtheme 2.2), and to requesting reasonable adjustments to aid listening, in both formal (e.g., educational) and informal settings:

*“If we’re at parties I am always turning the music down, I’m like, ‘I can't hear you, shut this noise up!’”*

Selected *Technology* (Subtheme 6.5) is also frequently reported as beneficial:

*“if we’re out at a restaurant typically I’ll wear high fidelity earplugs (…) So I can hear him talk to me, but it lessens the background noise.”*

*“When I watch tv, I have subtitles on all the time.”*

Technological solutions can also be used to avoid excessively difficult listening situations entirely, as is reported in relation to higher education by three participants:

*“I have a masters, I did it online with Penn state. I did it all online, so all I had to do was listen to videos and stuff, so I could do that when it was quiet in my own home.”*

A final common coping mechanism is *Withdrawal/avoidance* (Subtheme 6.6). This can be an adaptive and pre-emptive strategy, limiting time in a challenging listening environment, or building in sensory ‘down-time’ afterwards:

*“we have big family gatherings and everybody's talking and everything and I can only go for so long, and I’m like ‘right, we’ve gotta go’, we have to leave. Then afterwards I’m exhausted, and I have to retreat and go to a quiet place and just **be** for a little while.”*

However, reflexive ‘snapping’ or uncontrolled avoidance can also emerge, especially when no other solution is available.

*“not this most recent job I had but the one before that (…) there was no quiet room dedicated. There was an alcove near one of the elevators – it was still loud in there but it was quieter than everywhere else. And I’d go in there and cry sometimes.”*

## Discussion

The sensory experiences of autistic people are ostensibly well represented in the qualitative research literature but consideration of the auditory domain has been fairly limited. Resulting data have tended to focus on affective responses to sound (Ashburner et al., 2013; Jones et al., 2003; Kirby et al., 2015; Robertson & Simmons, 2015, 2018), though Sturrock et al. (2021) uncovered preliminary indicators that subtle listening difficulties could contribute to social, communicative, and emotional difficulties. To the authors’ knowledge, no peer-reviewed publication has reported in-depth qualitative data on the speech-perception experiences of autistic people. This appears an oversight worth correcting, based on the rich findings of the present study.

Among our autistic participants, presence of self-perceived speech-perception anomalies was universal. Every interviewee reported aspects of speech perception that they felt differed substantially from those around them. These predominantly took the form of perceptual *difficulties* in the presence of competing sounds, although heightened auditory perception for quiet sounds was also reported. Participants were clear that speech-perception difficulties are not just a side effect of social interaction difficulties; the two are distinct, though they can add and interact, with speech-perception difficulties exacerbating social difficulties and vice versa. The data also defy the notion that auditory impairment in the autistic population takes a single form; reported auditory anomalies were of several contrasting types. Each was well supported by data from a number of participants, though it is important to note that descriptions of experiences or phenomena were occasionally ambiguous, meaning that accurate determination of code frequencies was not feasible. Despite the inherent limitations of self-report data, we argue that the reported phenomena deserve investigation via adequately powered confirmatory research, taking care to disentangle the various forms of listening difficulty where possible, and ideally exploring heterogeneity within the autistic population via large samples.

It is clear, however, that the impact of listening difficulty on an individual is determined by factors besides *degree* of difficulty (see Figure 2). In particular, it seems that much of the consequent distress and disability results from *lack of awareness and understanding* of auditory differences: by communication partners, by institutions, by the autistic individuals themselves, and – perhaps most strikingly – by clinicians. Time and again, participants described having felt bewilderment and despondency in earlier life, when they had no explanation for their difficulties hearing in common listening environments, and no reliable strategies for handling them, even after summoning the courage to consult a clinician. We believe that raising awareness and understanding of auditory differences experienced by the autistic community will benefit its members by aiding self-knowledge, self-help and self-advocacy, but also by compelling wider society and institutions to adjust to the communication needs of some autistic citizens.

At present, without such measures in place, listening difficulties have diverse and substantial impacts, most prominently on socialising and emotional state. We consider it crucial that many participants expressed a desire for greater social participation, thwarted by the difficulty of following conversation in common social environments. Even in situations with just enough auditory information available to allow comprehension, effortful listening seems to take a toll, draining mental resources away from the ultimate goals of listening (comprehension, reflection, and retention) and sometimes exhausting the individual so badly that the cost of social participation becomes too high to justify. In a population whose social relations can be limited by various factors, reducing barriers caused by auditory difficulties could represent a relatively ‘easy win’ towards enhancing social opportunity. Our team was also struck by how visceral and far-reaching the emotional consequences of listening difficulty were for some interviewees. We believe that the social and emotional impacts of unmanaged listening difficulties in autistic individuals cannot be ignored, given the potential for interaction with coexisting social difficulties and given the prevalence of secondary mental health conditions in this population.

Though researchers must naturally strive to understand the causes of speech-perception difficulties, our data also suggest that more immediate benefit might be obtained by harnessing the insights and advice of the autistic community. Our small sample of nine interviewees described a wide array of strategies used to cope with listening difficulties, which were often hard won, developed through arduous experience, often in adulthood, generally without the aid of clinicians. A more comprehensive collection of such insights might be usefully developed into valuable self-help and clinical materials, and perhaps also form the basis for guidance for communication partners and institutions.

An important limitation of the present study is the small sample size. Although data appeared saturated by Analysis Meeting 4, and many themes were strongly supported, we cannot be certain that the experiences and views of our nine participants are representative of the wider autistic community. It is also likely that our recruitment and data-collection methods introduced sampling bias. Most participants hailed from online groups and forums, potentially selecting against individuals who dislike online social interactions, or who have faced online bullying or harassment, or find written communication challenging or aversive. Several participants learned of the study via Reddit.com, whose user base skews young, male, and educated. Our advertising stated that we wished to understand ‘how autistic and neurotypical people manage to hear what people are saying in noisy environments’, and although this wording was intended to be neutral, emphasising neither listening difficulties nor listening abilities, it is plausible that individuals with listening difficulties were especially keen to participate. The use of video-conferencing software to conduct the interviews surely selected against individuals uncomfortable with this medium, potentially leading us to underestimate the challenges posed by broadcast and telecommunications audio.

A further limitation is the study's lack of clinical hearing assessment, due to internet-based data collection. We cannot be certain that our sample did not include hearing-impaired participants. Clinical hearing loss – characterised by reduced sensitivity to quiet sounds – is relatively rare in this age range but may be much more common in autistic than in neurotypical young adults (Rydzewska et al., 2019). This raises another important issue: potential interactions in the autistic population between speech-perception deficits and additional clinical hearing loss. Had we deliberately recruited individuals with hearing loss, or simply recruited an older cohort, we might have observed far greater speech-perception difficulties.

Finally, we must emphasise to readers – and especially to researchers – that the present project has cemented our belief that genuine partnership between scientists and the autistic community is not only highly achievable but essential for conducting internally and externally valid research in this field. Throughout the lifecycle of our project, neurotypical members of our project team have been prompted to recognise and revise their assumptions, which might otherwise have posed risks to the aims of the research; this has been a discomfiting experience, but a valuable one.

## Conclusion

The first in-depth qualitative investigation of autistic speech-perception experiences has revealed diverse and widespread speech-perception anomalies – primarily listening difficulties – in a small sample of autistic adults. These can combine with other internal, interpersonal, and societal factors to induce profound impacts. Well designed, adequately powered, quantitative auditory research is needed to identify and disentangle the causes of impaired speech perception. The coping strategies developed by affected individuals could form the basis for self-help and clinical materials. More fundamental, however, is the need to combat lack of awareness of autistic listening difficulties – a phenomenon that appears pivotal in determining negative impacts – through outreach to a diversity of groups: the autistic community, institutions, communication partners, and clinicians.

## Supplemental Material

sj-docx-1-dli-10.1177_23969415221077532 - Supplemental material for Chasing the conversation: Autistic experiences of speech perception Click here for additional data file. Supplemental material, sj-docx-1-dli-10.1177_23969415221077532 for Chasing the conversation: Autistic experiences of speech perception by Alexandra Sturrock, Hannah Guest, Graham Hanks, George Bendo, Christopher J Plack and Emma Gowen in Autism & Developmental Language Impairments
