# Supplementary material for: Chasing the conversation: Autistic experiences of speech perception
Source: Autism Dev Lang Impair. 2022 Feb 24;7:23969415221077532. doi: 10.1177/23969415221077532 (PMC9620672; doi:10.1177/23969415221077532)
Supplement: sj-docx-1-dli-10.1177_23969415221077532 - Supplemental material for Chasing the conversation: Autistic experiences of speech perception [file sj-docx-1-dli-10.1177_23969415221077532.docx]

**SM1: Interview schedule**

Introduction

We want to find out about your experiences of **hearing speech** (i.e., hearing what people are saying). In life, there are all kinds of situations where we need to **hear and understand** what’s being said, e.g., at school, in work, or when socialising. Some situations can make it harder to hear than others. We’re going to ask you some questions to find out about your **hearing abilities**, and if there’s anything that affects them.

|  | No prompt | With prompt |
| --- | --- | --- |
| **1. Hearing abilities (and modifying factors)** | | |
| 1a. How easy or difficult do you find it to **hear and understand what people are saying**? | | |
| 1b. Are there any situations that **make it harder** to hear? | | |
| 1c. Are there any situations that **make it easier** to hear? | | |
| Follow-up prompts (if not already covered): | | |
| - Can you think of **specific situations** where you struggled with hearing? |  |  |
| - How do you find hearing in **social situations**? |  |  |
| - How do you find hearing in **education and/or work**? |  |  |
| - Does the **number of people** around you make a difference? |  |  |
| - Does the **loudness** of the situation make a difference? |  |  |
| - If there are background sounds, do the **types of sounds** make a difference? |  |  |
| - Are there **other sensory stimuli** that make a difference? |  |  |
| - Does **anything else** affect your hearing ability? |  |  |
|  | | |
| **2. Response to hearing difficulties** | | |
| 2a. Are there any **strategies** you use to make hearing easier? | | |
| 2b. In situations where hearing is difficult for you, does this ever affect **how you feel** or **what you do**? | | |
| 2c. If you have hearing difficulties, how have they **affected your life**? | | |
| Follow-up questions (if not already covered): | | |
| - Have hearing difficulties affected how you **feel about yourself**? |  |  |
| - Have hearing difficulties affected how you think **other people see you**? |  |  |
| - Have hearing difficulties affected your **education or employment**? |  |  |
| - Have hearing difficulties affected your **social life and relationships**? |  |  |
| - In general, have hearing difficulties **stopped you from doing anything**? |  |  |
| - Do you feel you having to put in **great effort** to hear clearly? Does this affect how you feel afterwards? |  |  |
|  | | |
| **3. General thoughts** | | |
| 3a. Do you think a study on hearing abilities in autistic people is a **good idea**? | | |
| 3b. If so, is there anything you think **we should be investigating** that we haven’t already mentioned? | | |
| 3c. Are you interested in possible **strategies or tools** to help you hear more easily? | | |
| 3d. Is there **anything else** you’d like to mention? | | |
